# Trends in Endoscopist Reporting Rates of Eosinophilic Gastrointestinal Diseases in Japan Evaluated by the Japan Endoscopy Database Project

**DOI:** 10.1002/deo2.70214

**Published:** 2025-09-26

**Authors:** Kazuya Miyaguchi, Yoshikazu Tsuzuki, Akiko Shiotani, Kiyohito Tanaka, Hiroyuki Imaeda

**Affiliations:** ^1^ Department of Gastroenterology Saitama Medical University Saitama Japan; ^2^ Department of Gastroenterology Kawasaki Medical University Okayama Japan; ^3^ Department of Gastroenterology Kyoto Second Red Cross Hospital Kyoto Japan

**Keywords:** endoscopic examinations, eosinophilic esophagitis, eosinophilic gastrointestinal diseases, *Helicobacter pylori*, Japanese Endoscopy Database

## Abstract

**Objectives:**

To examine the annual trends in the occurrence of eosinophilic gastrointestinal disease (EGID) in Japan.

**Methods:**

This study examined data from patients with EGID who underwent endoscopy at 716 facilities between January 2015 and March 2023. Data extracted from the Japanese Endoscopy Database (JED) included endoscopic procedure counts, patient demographics, *Helicobacter pylori* infection status, comorbidities, and balloon dilatation. Analyses incorporated both confirmed and suspected EGID cases.

**Results:**

In total, 9,940,870 endoscopic procedures were performed, identifying 9669 cases of EGID. From 2015 to 2023, the Cochran–Armitage trend test showed a significant increase in cases of eosinophilic esophagitis (EoE), eosinophilic gastritis (EoG), and eosinophilic duodenitis (EoD) (*p* < 0.001). However, no significant trend was observed for eosinophilic enteritis (EoN) or eosinophilic colitis (EoC) (*p* = 0.136). The number of EoE cases increased, with reporting rates changing from 36 (0.0464%) in 2015 to 3631 (0.176%) in 2022 (*p* < 0.001). EoG + EoD cases increased from one (0.0013%) to 410 (0.0199%) (*p* < 0.001), while EoN + EoC cases remained stable (*p* = 0.136).

**Conclusions:**

The JED project data showed increasing detection of EoE, EoG, and EoD in Japan, while EoN and/or EoC cases remained stable. These findings are based on reporting rates from individuals undergoing endoscopy, rather than estimates from population‐based endoscopist reporting rates.

## Graphical abstract

We analyzed 9,940,870 endoscopic procedures from the Japanese Endoscopy Database (2015–2023), identifying 9,669 eosinophilic gastrointestinal disease (EGID) cases. The prevalence of eosinophilic esophagitis, gastritis, and duodenitis increased significantly (*p* < 0.001), while enteritis and colitis remained unchanged (*p* = 0.136). EoE rose from 36 cases (0.0464%) in 2015 to 3631 (0.176%) in 2022. These findings indicate rising detection of upper gastrointestinal EGIDs in Japan, based on endoscopy reporting rates rather than population prevalence.

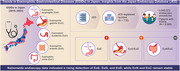

## 1 Introduction

Eosinophilic gastrointestinal diseases (EGIDs) are a group of chronic inflammatory conditions characterized by abnormal eosinophilic infiltration of the gastrointestinal (GI) tract, resulting in tissue inflammation, damage, and dysfunction [1]. Eosinophilic esophagitis (EoE) is defined by eosinophil infiltration in the esophageal mucosal epithelium, causing esophageal dysfunction. Eosinophilic gastroenteritis (EGE) involves eosinophilic infiltration with dysfunction in the stomach, small intestine, and/or colon, with or without esophageal involvement, and represents a common form of gastroenteritis. When limited to the large intestine, it is called eosinophilic colitis (EoC) [2]. Clinically, eosinophilic gastritis (EoG), eosinophilic duodenitis (EoD), eosinophilic enteritis (EoN), and EoC are classified by inflammation site. Non‐EoE EGID denotes eosinophilic infiltration of the GI tract, excluding the esophagus, including EoG, EoD, EoN, and EoC [3]. Recently, an increase in reported cases of eosinophilic esophagitis (EoE) has been observed in Japan, consistent with trends in other Asian countries based on questionnaire‐based surveys [4]. However, compared with Europe and the United States, the incidence of EoE remains lower in Japan, and the disease is frequently detected incidentally during endoscopic examinations [5, 6, 7, 8, 9].

The prevalence of EGIDs varies substantially between Western countries and Japan. In Europe and North America, the prevalence of EoE is estimated at 20–50 per 100,000 individuals, with a male‐to‐female ratio of approximately 2:1 to 3:1 [10]. In contrast, Japanese studies report an EoE prevalence ranging from 2.8 to 10.7 per 100,000 individuals [11], with detection rates of 0.02% to 0.4% among patients undergoing endoscopy [12]. Recent studies suggest that the incidence of EoE in Japan is increasing, mirroring trends observed in Western countries in the early 2000s. Data on non‐EoE EGIDs remain limited, with global prevalence estimates ranging from two to 28 per 100,000 individuals [13].

Epidemiological studies on EGE remain limited. The reported prevalence in Europe and the United States is approximately 18 cases per 100,000 persons (0.018%) [14]. Conversely, a meta‐analysis of 11 studies involving 13,377 patients with GI symptoms found that non‐EoE EGID occurred in 1.9% of patients [15]. Notably, EoE has been increasingly recognized and treated in Japan, with a reported 5.5‐fold increase in cases compared with EGE cases in 2011 [11]. Nevertheless, comprehensive epidemiological data on EGID in Japan remain scarce. Recent studies using large health insurance claims databases have provided valuable insights into EGID trends in Japan [12, 13]. While several studies using the Japanese Endoscopy Database (JED) have investigated the prevalence of *H. pylori* infection and gastric mucosal atrophy in patients with gastric cancer at the national level [16], to the best of our knowledge, this is the first large‐scale epidemiological study utilizing the JED to examine EGIDs.

## 2 Methods

### 2.1 Data Source

This study used data from the “Database Construction of Diseases and Treatment Procedures Related to Gastrointestinal Endoscopy,” a JED Project component led by the Japanese Society of Gastrointestinal Endoscopy. Data collection began at selected universities and high‐volume centers between 2015 and 2016. Since 2020, JED participation has become mandatory for educational facilities accredited by the Japanese specialist system, significantly expanding case registration.

### 2.2 Study Design and Population

This retrospective study included patients diagnosed with EGIDs (EoE, EoG, EoD, EoN, and EoC) who underwent endoscopy between January 1, 2015, and March 31, 2023. Both confirmed diagnoses and suspected cases based on clinical and endoscopic findings were included.

### 2.3 Patient Selection and Diagnostic Criteria

Patients were identified in the JED using EGID diagnostic codes through a four‐step process: (1) identification of all endoscopic procedures during the study period; (2) screening for EGID‐related diagnostic codes; (3) extraction of demographic and clinical data for qualifying patients; and (4) categorization by EGID subtype and anatomical location. Diagnoses were based on the clinical judgment of endoscopists at participating institutions, and the extent of histological confirmation varied. When a biopsy was not performed or was not feasible, EGID was recorded as suspected based on endoscopic findings, clinical course, and blood test results. Definitive diagnoses were those confirmed by biopsy following published criteria [3, 17].

### 2.4 Survey Endpoints

The study had three objectives. First, we conducted a comprehensive trend analysis of EGIDs over time, which included suspected cases and endoscopist reporting rates for definitive diagnoses. Second, we assessed the number of patients with EGIDs by geographic region, specifically examining prefecture‐level distributions across Japan in 2022. Third, for the patients identified with EGIDs, we evaluated the annual number of cases through comparisons across fiscal years; demographic and clinical characteristics, including age, sex, smoking and drinking histories, and *Helicobacter pylori* infection status; and the presence of comorbid conditions such as hiatal hernia, reflux esophagitis, atrophic gastritis, and gastric or duodenal ulcers. Our findings do not present definitive diagnoses but rather reflect the endoscopist reporting rate of both suspected and confirmed cases of EGID based on the JED database.

The study protocol adhered to the revised Declaration of Helsinki (1989) and was approved by the institutional review board (Approval No.: 2023–064). The Japan Endoscopy Society coordinated the ethical review. Most participating facilities used an opt‐out approach, while those without opt‐out procedures obtained informed consent according to institutional protocols. We additionally investigated the 2022 regional distribution of patients with EGID, identifying prefectures with the highest diagnosed case numbers.

### 2.5 Rationale and Assumptions for Regional Analysis

Several methodological considerations support the selection of 2022 for the prefecture‐level analysis:

1. Data completeness: The year 2022 represents the most recent calendar year with full participation of JED‐registered facilities (*N* = 716), ensuring comprehensive geographic coverage.
2. Diagnostic stability: By 2022, clinical recognition of EGIDs among Japanese gastroenterologists had reached a more stable level, thereby reducing potential confounding effects associated with the rapid evolution of diagnostic practices in earlier years.
3. Minimization of coronavirus disease 2019 (COVID‐19) impact: Between 2020 and 2021, the COVID‐19 pandemic significantly disrupted routine endoscopic examinations. By 2022, however, endoscopic screening and diagnostic practices had largely returned to pre‐pandemic levels.

### 2.6 Statistical Analyses

Continuous data are reported as mean ± standard deviation for normally distributed variables. Categorical data are presented as counts and percentages. Between‐group comparisons across years were performed using a linear contrast test within an analysis of variance framework for continuous variables and the Cochran–Armitage trend test for categorical variables. Bonferroni correction was applied to adjust for multiple comparisons. Statistical analyses were performed using SPSS version 29.0.2.0 for Windows (IBM Japan, Tokyo, Japan), with *p* < 0.05 considered statistically significant.

## 3 Results

Overall, 716 facilities were registered in the JED. Across these institutions, 9,940,870 endoscopic procedures were recorded, including 6,859,907 esophagogastroduodenoscopies (EGDs) and 3,080,963 lower GI endoscopies, comprising small bowel endoscopy and colonoscopy. Of these, 20,846 endoscopic procedures were performed for EGIDs, and 9669 patients with EGIDs were included in the analysis.

In total, 14,813 EGID lesions were identified across all anatomical sites, exceeding the number of patients because some individuals had multiple lesions at different locations.

Among the 9669 patients with EGIDs, both histologically confirmed and suspected cases were included, based on endoscopic findings and clinical presentation in accordance with institutional diagnostic criteria. The proportion of suspected versus confirmed cases was approximately 30% among all EGID subtypes, although this varied by institution and disease subtype.

### 3.1 Trend Analysis Over Time and Aggregation by Prefecture

Overall, 14,813 EGID lesions were identified, and the trend analysis was performed over the last 8 years (2015–2023). The sharp decrease in the number of endoscopic examinations in 2023 reflects the short survey period, which includes only the first quarter of the year (January to March 2023) (Table 1).

**TABLE 1:** Endoscopy of trend analysis: 2015–2023 (Cochran–Armitage trend test).

	2015	2016	2017	2018	2019	2020	2021	2022	2023*	*p*‐Value
EGD										
Total	77,602	95,252	349,616	151,139	383,331	1,301,158	2,061,877	2,059,831	380,101	
EoE	35	43	209	142	459	1,638	2,470	2,613	437	**<0.001**
%	0.0464%	0.0577%	0.0644%	0.1237%	0.1422%	0.1459%	0.1561%	0.1763%	0.1644%	
EGD										
Total	77,602	95,252	349,616	151,139	383,331	1,301,158	2,061,877	2,059,831	380,101	
EoG and/or EoD	3	8	55	13	96	352	528	482	65	**<0.001**
%	0.0013%	0.0084%	0.0126%	0.0119%	0.0243%	0.0191%	0.0208%	0.0199%	0.0184%	
CS+DBE										
Total	37,954	43,221	138,471	67,922	176,261	616,491	949,877	885,831	164,935	
EoN and/or EoC	4	11	53	16	82	301	436	325	35	0.136
%	0.0053%	0.0255%	0.0209%	0.0177%	0.0136%	0.0248%	0.0256%	0.0220%	0.0212%	

Data are presented as numbers or percentages.

*p*‐Value: Cochran–Armitage trend test.

Abbreviations: CS, colonoscopy; DBE, double balloon endoscopy; EGD, esophagogastroduodenoscopy; EoC, eosinophilic colitis; EoD, eosinophilic duodenitis; EoE, eosinophilic esophagitis; EoG, eosinophilic gastritis; EoN, eosinophilic enteritis.

* The 2023 survey will be conducted over a 3‐month period.

We also conducted a trend analysis of confirmed EGID cases: all EGIDs showed a significant increase over time (Table 2).

**TABLE 2:** Confirmed cases of trend analysis: 2015–2023 (Cochran–Armitage trend test).

	2015	2016	2017	2018	2019	2020	2021	2022	2023*	*p*‐Value
EoE										**<0.001**
Total	35	43	209	142	459	1,638	2,470	2,613	437	
Confirmed	20	25	127	90	265	1079	1641	1721	281	
%	57.1	58.1	60.8	63.4	57.7	65.9	66.4	65.9	64.3	
EoG and/or EoD										**<0.001**
Total	3	8	55	13	96	352	528	482	65	
Confirmed	2	6	36	7	50	223	356	324	39	
%	66.7	75.0	65.5	53.8	52.1	63.4	67.4	67.2	60.0	
EoN and/or EoC										**<0.001**
Total	4	11	53	16	82	301	436	325	49	
Confirmed	2	10	36	10	59	156	238	167	28	
%	50	90.9	67.9	62.5	72.0	51.8	54.6	51.4	57.1	

Data are presented as numbers or percentages.

p‐Value: Cochran–Armitage trend test.

Abbreviations: EGD, esophagogastroduodenoscopy; EoC, eosinophilic colitis; EoD, eosinophilic duodenitis; EoE, eosinophilic esophagitis; EoG, eosinophilic gastritis; EoN, eosinophilic enteritis.

* The 2023 survey will be conducted over a 3‐month period.

### 3.2 EoE

**FIGURE 1:**
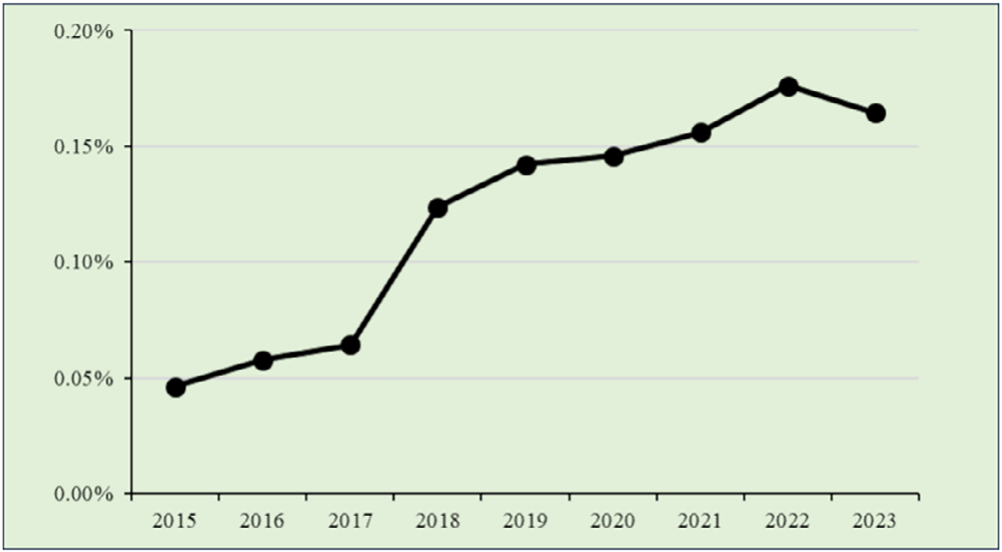
Eosinophilic esophagitis cases/total cases.

EoE accounted for approximately 0.1% of all endoscopic procedures, with a significant upward trend over time (p < 0.001) (Figure 1). The highest EoE‐to‐EGD detection ratio was observed in 2022, reaching 0.176% (*N* = 3631).

### 3.3 EoG and/or EoD

**FIGURE 2:**
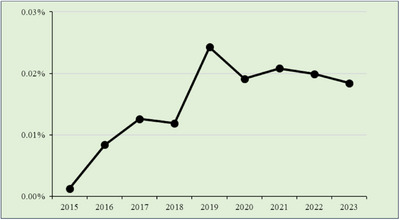
Eosinophilic gastritis and/or eosinophilic duodenitis/total cases.

EoG and EoD were analyzed together due to their similar temporal trends, despite differences in anatomical location (Figure 2). Their combined detection rates were lower than those of EoE, ranging from 0.01% to 0.02%. The highest combined EoG + EoD‐to‐EGD detection ratio was 0.0243% in 2019, with a corresponding detection of 429 cases in 2021 (0.0208%). A significant increasing trend was observed (*p* < 0.001).

### 3.4 EoN and/or EoC

**FIGURE 3:**
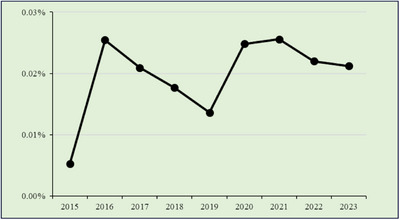
Eosinophilic colitis cases/total cases.

EoN and EoC were jointly analyzed because both affect the lower GI tract and exhibited similar temporal patterns (Figure 3). The highest combined endoscopist reporting rate, calculated as (EoN + EoC) divided by the total number of colonoscopies (CS) and double‐balloon endoscopies (DBE), was 0.0256% in 2021 (*N* = 243), with a reporting rate of 0.022% in 2022. No significant temporal trend was observed (*p* = 0.136).

**FIGURE 4:**
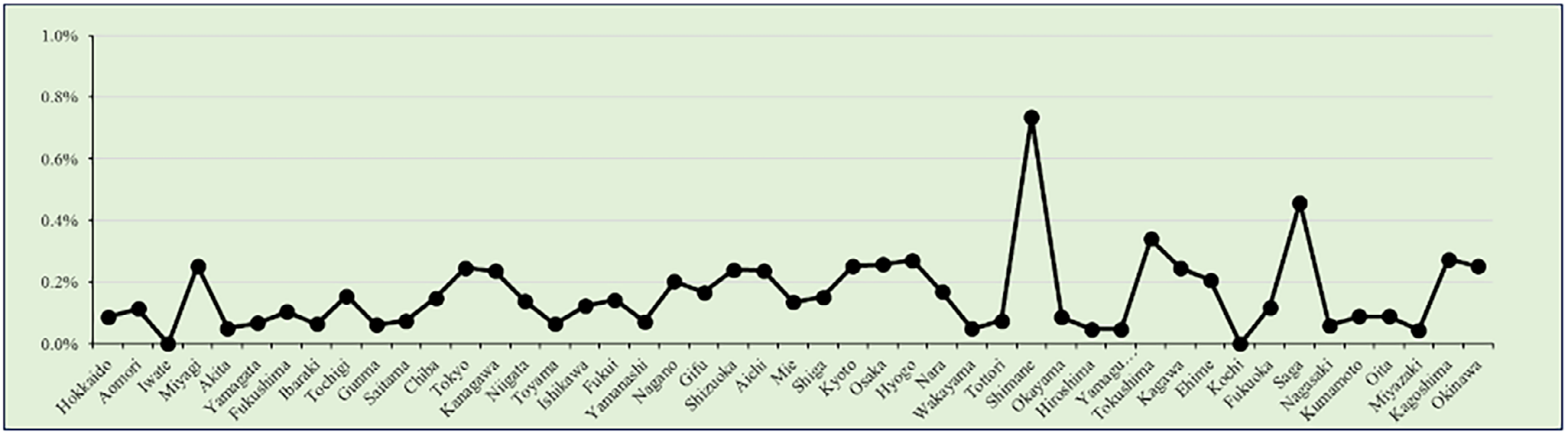
Eosinophilic esophagitis cases/regional aggregation by prefecture.

**FIGURE 5:**
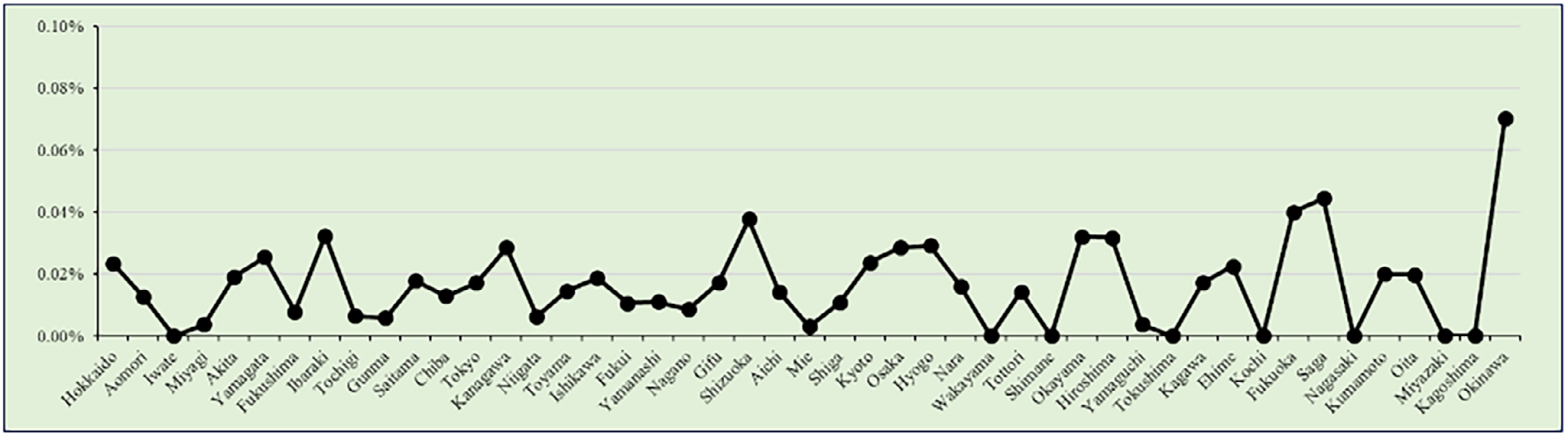
Eosinophilic gastritis and eosinophilic duodenitis/regional aggregation by prefecture.

**FIGURE 6:**
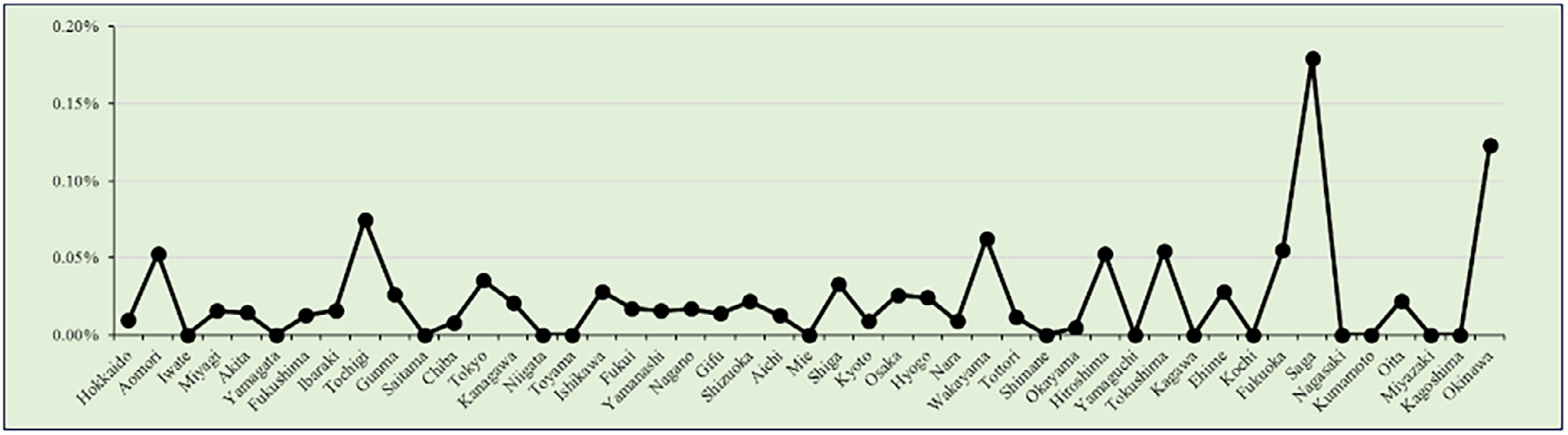
Eosinophilic enteritis and/or eosinophilic colitis cases/regional aggregation by prefecture.

Regional aggregation by prefecture is presented in Table S1. Comparisons are based on data from 2022, the year with the highest number of EGID cases (Figures 4, 5, 6).

### 3.5 Patient Demographics and Clinical Characteristics

Table S2 summarizes the patient background characteristics for EGIDs. The most frequently diagnosed subtype during the study period was EoE (84.9%), followed by non‐EoE‐EGID (15.7%).

Table S2‐A presents a trend analysis of EoE. In 2022 (N = 2613), the mean age of the patients was 52.0 ± 14.6 years; the cohort included 1789 males and 822 females. No significant temporal trends were observed in age, sex distribution, or smoking history. However, a significant downward trend was noted in the number of patients testing negative for *H. pylori*, accompanied by a significant increase in the number of patients who had not been tested. The high proportion of untested individuals (approximately 40%) limits the interpretability of any association between *H. pylori* infection status and EGIDs.

Table S1‐B presents a trend analysis of non‐EoE‐EGID. The highest number of cases was recorded in 2021 (*N* = 612), comprising 369 males and 326 females, with a mean age of 51.0 ± 19.3 years. As with EoE, there were no significant trends in age, sex, or smoking history. A significant downward trend was observed in the number of *H. pylori*‐negative cases.

## 4 Discussion

To the best of our knowledge, this study represents the largest analysis of EGID detection rates in Japan using endoscopic database data. EoE diagnosis has increased in Japan in recent years, with detection rates expected to rise further [18]. Using JED data, our 8‐year trend analysis showed that EoE accounted for approximately 0.1% of all endoscopic examinations, with the highest detection rate reaching 0.176% per EGD.

EoE diagnoses in Japan have rapidly increased, partly reflecting a true disease incidence rise. A 2004–2009 survey showed that EoE comprised 15% of all EGID cases, with eosinophilic gastroenteritis accounting for 85% [11]. Our findings showed that EoE cases represented 0.2% of all endoscopic procedures, consistent with previous reports. The 2022 EoE group included 1,789 males and 822 females (mean age 52.0 ± 14.6 years), while the 2021 non‐EoE‐EGID group included 369 males and 326 females (mean age 51.0 ± 19.3 years), showing no significant age difference between groups. EoE occurred significantly more frequently in males, while non‐EoE‐EGID showed a balanced sex ratio, demonstrating a higher male proportion in EoE than in non‐EoE‐EGID.

The observed increase in EoE incidence may be attributed to several factors, including the rising prevalence of coexisting allergic diseases [19, 20, 21] and the decline in *H. pylori* infection rates [22]. Additionally, sex‐based differences in EoE incidence are likely associated with endogenous factors, such as estrogen levels [23]. The increasing recognition of characteristic endoscopic features of EoE, such as esophageal furrows, may also have contributed to improved detection. However, during the relatively short observation period of this study, only minimal changes were observed in *H. pylori* infection rates among patients with EGIDs.

A strong correlation has been reported between *H. pylori* infection and regional water supply and wastewater penetration rates, suggesting that improved sanitation directly influences *H. pylori* colonization. The prevalence of *H. pylori* infection in Japan was approximately 27% in 2016 and is projected to decline to 5% by 2050 [24].

In this study, EoE appeared to be most common in Shimane (*N* = 18), Saga (*N* = 31), and Tokushima (*N* = 22; 0.3398%) Prefectures. However, despite these figures, significant regional differences in EoE prevalence are unlikely. The increase in EGIDs is expected to continue as *H. pylori* infection rates decline [24]. Several studies conducted in Europe, the United States, and Japan have reported significantly lower infection rates in patients with EoE than in non‐EoE control groups [25, 26].

Analysis of data from approximately 377,000 individuals demonstrated that H. pylori exposure was associated with a 37% reduction in the likelihood of developing EoE. This association remained consistent across regions, age groups, and levels of *H. pylori* prevalence [27].

Conversely, more recent studies suggest that the *H. pylori* infection rate in patients with EoE does not significantly differ from that in non‐EoE populations, implying that environmental changes associated with *H. pylori* exposure, rather than immunological effects of infection suppression, may more strongly contribute to EoE development [28]. In the present study, a decrease in the number of patients testing negative for *H. pylori* was observed alongside an increase in EoE cases; however, the proportion of untested individuals also increased, complicating the interpretation of this association.

Potential environmental risk factors for EoE include exposure to pollen, climate variability, and population density (e.g., rural vs. urban settings) [29].

We initially hypothesized that regional environmental and medical factors might influence EGID detection rates. However, regional differences observed in our analysis were more likely to be attributable to diagnostic ability‐related factors rather than to true epidemiological variation. Elevated detection rates in specific prefectures may reflect the increased regional clinical interest in EGID, more proactive diagnostic practices, or improved access to specialized facilities capable of establishing a diagnosis.

In our study, the number of non‐EoE EGID cases indicated a low incidence of EoG and/or EoD (0.01%–0.02%), with a peak combined reporting rate (EoG and/or EoD per EGD) of 0.0243% observed in 2019. As non‐EoE‐EGIDs lack specific endoscopic features, they are challenging to identify via endoscopy alone without clinical context, a limitation noted in previous studies. However, improved recognition of relevant findings by experienced endoscopists may increase detection rates.

Okinawa Prefecture showed the highest (EoG + EoD)/EGD ratio (0.07%) in 2022, although no consistent regional differences were identified across Japan. Saga Prefecture recorded the highest [(EoN + EoC)/(CS + DBE)] percentage (0.18%), followed by Okinawa and Tochigi Prefectures, although the underlying reasons remain unclear. Given non‐EoE‐EGID's chronic nature and nonspecific presentation, fundamental disease‐modifying treatments are needed beyond symptomatic management.

### 4.1 Methodological Considerations and Limitations

This study has some limitations. First, the duration of this survey was shortened to 3 months in 2023. Second, the JED database primarily records diagnostic codes related to endoscopic procedures but does not systematically document histological findings or standardized eosinophil counts. Consequently, many cases classified as “non‐EoE EGID” may reflect clinical suspicion based on endoscopic findings or symptoms rather than a definitive diagnosis, including histological criteria. Because cases of non‐EoE EGID often lack characteristic endoscopic features and their diagnosis relies heavily on tissue eosinophilia for diagnosis, this limitation is particularly significant. Third, varying diagnostic criteria across institutions may have affected regional comparisons and temporal trend analyses. Fourth, regarding the presence or absence of *H. pylori*, in Japan, insurance covers eradication therapy for patients with chronic gastritis, and testing for *H. pylori* is widely available. However, this contradicts the finding that only 40% of patients in this study underwent *H. pylori* testing. This discrepancy may be due to the participation of multiple facilities; in many cases, the presence or absence of *H. pylori* testing was not consistently recorded. Variability in testing practices across institutions may have contributed to the substantial proportion of untested cases. Moreover, for patients undergoing their first EGD, H. pylori testing may have been performed after the endoscopy and JED registration, which could make the recorded testing rate appear low, even when endoscopic findings suggest H. pylori infection.

Fifth, prefecture‐specific analyses used relatively few cases, risking sampling bias. Sixth, the study included individuals undergoing endoscopy for clinical indications, and thus the results reflect detection rates among endoscopy patients, not population‐based prevalence. Seventh, JED's procedure‐based recording may duplicate data when patients undergo multiple endoscopies. Eighth, the expansion of JED participation after 2020, particularly among teaching hospitals, may have contributed to higher detection rates through improved recognition, diagnostic expertise, and changes in case mix. This does not fully account for structural changes in facility type or the addition of new centers. Consequently, temporal increases should be interpreted with caution.

In summary, this study used JED data to investigate nationwide trends in endoscopic detection of EGIDs in Japan. We observed increasing detection rates of EoE, EoG, and EoD, while EoN and EoC detection rates remained relatively unchanged. These findings represent detection rates among patients undergoing endoscopy and should not be interpreted as estimates of population‐level prevalence.

## Author Contributions

Manuscript drafting: Kazuya Miyaguchi. Statistical analysis: Kazuya Miyaguchi and Yoshikazu Tsuzuki. Supervision: Akiko Shiotani, Kiyohito Tanaka, and Hiroyuki Imaeda. The final version of the manuscript was read and approved by all authors.

## Ethics Statement

**Approval of the research protocol by an Institutional Review Board**: The study protocol was in accordance with the tenets of the revised Declaration of Helsinki (1989) and was approved by the appropriate institutional review board (2023–064).

## Consent

Written informed consent was obtained from all patients.

## Conflicts of Interest

The authors declare no conflicts of interest.

## Clinical Trial Registration

Registry and the Registration No. of the study/trial: 2023‐064.

## Supporting information




**TABLE S1** Prefecture‐by‐prefecture totals in 2022.

**TABLE S2** Comparison of eosinophilic gastrointestinal diseases (EGIDs) between years: trend analysis.
**Table S2‐A** Trend analysis: eosinophilic esophagitis (EoE).
**Table S2‐B** Trend analysis: non‐eosinophilic esophagitis, eosinophilic gastrointestinal diseases (non‐EoE EGID).

## Data Availability

The data that support the findings of this study are available from the corresponding author upon reasonable request.
