# Supplementary material for: Trends in Endoscopist Reporting Rates of Eosinophilic Gastrointestinal Diseases in Japan Evaluated by the Japan Endoscopy Database Project
Source: DEN Open. 2025 Sep 26;6(1):e70214. doi: 10.1002/deo2.70214 (PMC12474659; doi:10.1002/deo2.70214)
Supplement: Supplementary file 1 — TABLE S1 Prefecture‐by‐prefecture totals in 2022. [file DEO2-6-e70214-s002.docx]

Table S1. Prefecture-by-prefecture totals in 2022

|  | EGD | | | |  | EGD | | | |  | CS+DBE | | | |
| --- | --- | --- | --- | --- | --- | --- | --- | --- | --- | --- | --- | --- | --- | --- |
|  | Total | EoE | NonEoE | % |  | Total | Eosinophilic gastritis and eosinophilic duodenitis/ | Others | % |  | Total | EoN+EoC | NonEoN+EoC | % |
| Hokkaido | 77,129 | 68 | 77,061 | 0.0882% |  | 77,129 | 18 | 77,111 | 0.0233% |  | 42,000 | 4 | 41,996 | 0.0095% |
| Aomoir | 7,940 | 9 | 7,931 | 0.1134% |  | 7,940 | 1 | 7,939 | 0.0126% |  | 5,672 | 3 | 5,669 | 0.0529% |
| Iwate | 1,649 | 0 | 1,649 | 0.0000% |  | 1,649 | 0 | 1,649 | 0.0000% |  | 403 | 0 | 403 | 0.0000% |
| Miyagi | 26,444 | 67 | 26,377 | 0.2534% |  | 26,444 | 1 | 26,443 | 0.0038% |  | 12,712 | 2 | 12,710 | 0.0157% |
| Akita | 15,681 | 8 | 15,673 | 0.0510% |  | 15,681 | 3 | 15,678 | 0.0191% |  | 6,916 | 1 | 6,915 | 0.0145% |
| Yamagata | 11,767 | 8 | 11,759 | 0.0680% |  | 11,767 | 3 | 11,764 | 0.0255% |  | 7,699 | 0 | 7,699 | 0.0000% |
| Fukushima | 38,166 | 40 | 38,126 | 0.1048% |  | 38,166 | 3 | 38,163 | 0.0079% |  | 15,691 | 2 | 15,689 | 0.0127% |
| Ibaraki | 30,901 | 20 | 30,881 | 0.0647% |  | 30,901 | 10 | 30,891 | 0.0324% |  | 12,574 | 2 | 12,572 | 0.0159% |
| Tochigi | 15,481 | 24 | 15,457 | 0.1550% |  | 15,481 | 1 | 15,480 | 0.0065% |  | 9,346 | 7 | 9,339 | 0.0749% |
| Gunma | 51,220 | 32 | 51,188 | 0.0625% |  | 51,220 | 3 | 51,217 | 0.0059% |  | 15,351 | 4 | 15,347 | 0.0261% |
| Saitama | 73,022 | 55 | 72,967 | 0.0753% |  | 73,022 | 13 | 73,009 | 0.0178% |  | 40,985 | 0 | 40,985 | 0.0000% |
| Chiba | 100,295 | 149 | 100,146 | 0.1486% |  | 100,295 | 13 | 100,282 | 0.0130% |  | 49,526 | 4 | 49,522 | 0.0081% |
| Tokyo | 256,313 | 635 | 255,678 | 0.2477% |  | 256,313 | 44 | 256,269 | 0.0172% |  | 113,586 | 40 | 113,546 | 0.0352% |
| Kanagawa | 105,439 | 249 | 105,190 | 0.2362% |  | 105,439 | 30 | 105,409 | 0.0285% |  | 52,245 | 11 | 52,234 | 0.0211% |
| Niigata | 31,817 | 44 | 31,773 | 0.1383% |  | 31,817 | 2 | 31,815 | 0.0063% |  | 9,499 | 0 | 9,499 | 0.0000% |
| Toyama | 27,578 | 18 | 27,560 | 0.0653% |  | 27,578 | 4 | 27,574 | 0.0145% |  | 10,974 | 0 | 10,974 | 0.0000% |
| Ishikawa | 48,191 | 59 | 48,132 | 0.1224% |  | 48,191 | 9 | 48,182 | 0.0187% |  | 10,563 | 3 | 10,560 | 0.0284% |
| Fukui | 47,359 | 68 | 47,291 | 0.1436% |  | 47,359 | 5 | 47,354 | 0.0106% |  | 11,604 | 2 | 11,602 | 0.0172% |
| Yamanashi | 18,047 | 13 | 18,034 | 0.0720% |  | 18,047 | 2 | 18,045 | 0.0111% |  | 6,325 | 1 | 6,324 | 0.0158% |
| Nagano | 57,855 | 117 | 57,738 | 0.2022% |  | 57,855 | 5 | 57,850 | 0.0086% |  | 17,856 | 3 | 17,853 | 0.0168% |
| Gifu | 34,789 | 58 | 34,731 | 0.1667% |  | 34,789 | 6 | 34,783 | 0.0172% |  | 14,335 | 2 | 14,333 | 0.0140% |
| Shizuoka | 60,897 | 147 | 60,750 | 0.2414% |  | 60,897 | 23 | 60,874 | 0.0378% |  | 27,389 | 6 | 27,383 | 0.0219% |
| Aichi | 119,023 | 284 | 118,739 | 0.2386% |  | 119,023 | 17 | 119,006 | 0.0143% |  | 55,688 | 7 | 55,681 | 0.0126% |
| Mie | 31,803 | 43 | 31,760 | 0.1352% |  | 31,803 | 1 | 31,802 | 0.0031% |  | 13,366 | 0 | 13,366 | 0.0000% |
| Shiga | 27,741 | 42 | 27,699 | 0.1514% |  | 27,741 | 3 | 27,738 | 0.0108% |  | 12,151 | 4 | 12,147 | 0.0329% |
| Kyoto | 71,331 | 181 | 71,150 | 0.2537% |  | 71,331 | 17 | 71,314 | 0.0238% |  | 22,068 | 2 | 22,066 | 0.0091% |
| Osaka | 182,313 | 471 | 181,842 | 0.2583% |  | 182,313 | 52 | 182,261 | 0.0285% |  | 81,483 | 21 | 81,462 | 0.0258% |
| Hyogo | 82,351 | 223 | 82,128 | 0.2708% |  | 82,351 | 24 | 82,327 | 0.0291% |  | 41,010 | 10 | 41,000 | 0.0244% |
| Nara | 24,757 | 42 | 24,715 | 0.1696% |  | 24,757 | 4 | 24,753 | 0.0162% |  | 10,993 | 1 | 10,992 | 0.0091% |
| Wakayama | 18,427 | 9 | 18,418 | 0.0488% |  | 18,427 | 0 | 18,427 | 0.0000% |  | 4,783 | 3 | 4,780 | 0.0627% |
| Tottori | 28,046 | 21 | 28,025 | 0.0749% |  | 28,046 | 4 | 28,042 | 0.0143% |  | 8,799 | 1 | 8,798 | 0.0114% |
| Shimane | 2,443 | 18 | 2,425 | 0.7368% |  | 2,443 | 0 | 2,443 | 0.0000% |  | 530 | 0 | 530 | 0.0000% |
| Okayama | 59,451 | 51 | 59,400 | 0.0858% |  | 59,451 | 19 | 59,432 | 0.0320% |  | 20,747 | 1 | 20,746 | 0.0048% |
| Hiroshima | 47,334 | 23 | 47,311 | 0.0486% |  | 47,334 | 15 | 47,319 | 0.0317% |  | 22,893 | 12 | 22,881 | 0.0524% |
| Yamaguchi | 27,215 | 13 | 27,202 | 0.0478% |  | 27,215 | 1 | 27,214 | 0.0037% |  | 8,321 | 0 | 8,321 | 0.0000% |
| Tokushima | 6,474 | 22 | 6,452 | 0.3398% |  | 6,474 | 0 | 6,474 | 0.0000% |  | 1,828 | 1 | 1,827 | 0.0547% |
| Kagawa | 23,217 | 57 | 23,160 | 0.2455% |  | 23,217 | 4 | 23,213 | 0.0172% |  | 8,112 | 0 | 8,112 | 0.0000% |
| Ehime | 22,115 | 46 | 22,069 | 0.2080% |  | 22,115 | 5 | 22,110 | 0.0226% |  | 7,087 | 2 | 7,085 | 0.0282% |
| Kochi | 6,575 | 0 | 6,575 | 0.0000% |  | 6,575 | 0 | 6,575 | 0.0000% |  | 2,878 | 0 | 2,878 | 0.0000% |
| Fukuoka | 55,016 | 65 | 54,951 | 0.1181% |  | 55,016 | 22 | 54,994 | 0.0400% |  | 27,094 | 15 | 27,079 | 0.0554% |
| Saga | 6,757 | 31 | 6,726 | 0.4588% |  | 6,757 | 3 | 6,754 | 0.0444% |  | 3,340 | 6 | 3,334 | 0.1796% |
| Nagasaki | 13,349 | 8 | 13,341 | 0.0599% |  | 13,349 | 0 | 13,349 | 0.0000% |  | 4,362 | 0 | 4,362 | 0.0000% |
| Kumamoto | 30,084 | 27 | 30,057 | 0.0897% |  | 30,084 | 6 | 30,078 | 0.0199% |  | 16,729 | 0 | 16,729 | 0.0000% |
| Oita | 10,117 | 9 | 10,108 | 0.0890% |  | 10,117 | 2 | 10,115 | 0.0198% |  | 4,499 | 1 | 4,498 | 0.0222% |
| Miyazaki | 4,465 | 2 | 4,463 | 0.0448% |  | 4,465 | 0 | 4,465 | 0.0000% |  | 2,240 | 0 | 2,240 | 0.0000% |
| Kagoshima | 4,374 | 12 | 4,362 | 0.2743% |  | 4,374 | 0 | 4,374 | 0.0000% |  | 2,654 | 0 | 2,654 | 0.0000% |
| Okinawa | 17,073 | 43 | 17,030 | 0.2519% |  | 17,073 | 12 | 17,061 | 0.0703% |  | 8,925 | 11 | 8,914 | 0.1232% |

Data are presented as numbers. EGD, esophagogastroduodenoscopy; EoE, eosinophilic esophagitis; CS, colonoscopy; double balloon endoscopy; EoN, eosinophilic enteritis; EoC, eosinophilic colitis.
