# Supplementary material for: Trends in Endoscopist Reporting Rates of Eosinophilic Gastrointestinal Diseases in Japan Evaluated by the Japan Endoscopy Database Project
Source: DEN Open. 2025 Sep 26;6(1):e70214. doi: 10.1002/deo2.70214 (PMC12474659; doi:10.1002/deo2.70214)
Supplement: Supplementary file 2 — TABLE S2 Comparison of eosinophilic gastrointestinal diseases (EGIDs) between years: trend analysis. Table S2‐A Trend analysis: eosinophilic esophagitis (EoE). Table S2‐B Trend analysis: non‐eosinophilic esophagitis, eosinophilic gastrointestinal diseases (non‐EoE EGID). [file DEO2-6-e70214-s001.docx]

Table S2. Comparison of eosinophilic gastrointestinal diseases (EGIDs) between years: trend analysis

Table S2-A. Trend analysis: eosinophilic esophagitis (EoE)

|  |  | 2015 | | |  | 2016 | | |  | 2017 | | |  | 2018 | | |  | 2019 | | |  | 2020 | | |  | 2021 | | |  | 2022 | | |  | 2023* | | | P-value |  |
| --- | --- | --- | --- | --- | --- | --- | --- | --- | --- | --- | --- | --- | --- | --- | --- | --- | --- | --- | --- | --- | --- | --- | --- | --- | --- | --- | --- | --- | --- | --- | --- | --- | --- | --- | --- | --- | --- | --- |
|  |  | (n=35) | | |  | (n=43) | | |  | (n=209) | | |  | (n=142) | | |  | (n=459) | | |  | (n=1638) | | |  | (n=2470) | | |  | (n=2613) | | |  | (n=437) | | | for trend |  |
| Age | | 52.7 | ± | 15.2 |  | 54.0 | ± | 15.4 |  | 49.8 | ± | 15.7 |  | 51.0 | ± | 14.0 |  | 51.0 | ± | 16.1 |  | 51.4 | ± | 15.3 |  | 51.7 | ± | 15.4 |  | 52.0 | ± | 14.6 |  | 53.4 | ± | 14.7 | 0.952 | a |
| Sex | |  |  |  |  |  |  |  |  |  |  |  |  |  |  |  |  |  |  |  |  |  |  |  |  |  |  |  |  |  |  |  |  |  |  |  | 0.245 | b |
|  | Male | 29 | , | 82.9 |  | 35 | , | 81.4 |  | 154 | , | 73.7 |  | 98 | , | 69.0 |  | 305 | , | 66.4 |  | 1094 | , | 66.9 |  | 1676 | , | 67.9 |  | 1789 | , | 68.5 |  | 296 | , | 67.9 |  |  |
|  | Female | 6 | , | 17.1 |  | 8 | , | 18.6 |  | 55 | , | 26.3 |  | 44 | , | 31.0 |  | 154 | , | 33.6 |  | 542 | , | 33.1 |  | 792 | , | 32.1 |  | 822 | , | 31.5 |  | 140 | , | 32.1 |  |  |
|  | Others | 0 | , | 0.0 |  | 0 | , | 0.0 |  | 0 | , | 0.0 |  | 0 | , | 0.0 |  | 0 | , | 0.0 |  | 0 | , | 0.0 |  | 0 | , | 0.0 |  | 0 | , | 0.0 |  | 0 | , | 0.0 |  |  |
|  | Unknown | 0 |  |  |  | 0 |  |  |  | 0 |  |  |  | 0 |  |  |  | 0 |  |  |  | 2 |  |  |  | 2 |  |  |  | 2 |  |  |  | 1 |  |  |  |  |
| Smoking history | |  |  |  |  |  |  |  |  |  |  |  |  |  |  |  |  |  |  |  |  |  |  |  |  |  |  |  |  |  |  |  |  |  |  |  |  |  |
|  | Current smoker | 0 | , | 0.0 |  | 3 | , | 13.6 |  | 5 | , | 9.8 |  | 9 | , | 12.5 |  | 11 | , | 10.6 |  | 18 | , | 9.1 |  | 40 | , | 13.4 |  | 30 | , | 11.4 |  | 4 | , | 9.8 | 0.284 | b |
|  | Former Smoker | 11 | , | 40.7 |  | 9 | , | 40.9 |  | 25 | , | 49.0 |  | 20 | , | 27.8 |  | 33 | , | 31.7 |  | 45 | , | 22.8 |  | 86 | , | 28.9 |  | 81 | , | 30.8 |  | 17 | , | 41.5 | 0.160 | b |
|  | Non-smoker | 16 | , | 59.3 |  | 10 | , | 45.5 |  | 21 | , | 41.2 |  | 43 | , | 59.7 |  | 60 | , | 57.7 |  | 134 | , | 68.0 |  | 172 | , | 57.7 |  | 152 | , | 57.8 |  | 20 | , | 48.8 | 0.530 | b |
|  | Unknown | 8 |  |  |  | 21 |  |  |  | 158 |  |  |  | 70 |  |  |  | 355 |  |  |  | 1441 |  |  |  | 2172 |  |  |  | 2350 |  |  |  | 396 |  |  |  |  |
| History of alcohol | |  |  |  |  |  |  |  |  |  |  |  |  |  |  |  |  |  |  |  |  |  |  |  |  |  |  |  |  |  |  |  |  |  |  |  |  |  |
|  | Habitual drinker | 9 | , | 33.3 |  | 9 | , | 42.9 |  | 29 | , | 56.9 |  | 27 | , | 39.1 |  | 35 | , | 33.7 |  | 59 | , | 29.8 |  | 84 | , | 28.2 |  | 79 | , | 32.4 |  | 21 | , | 51.2 | 0.066 | b |
|  | Non-habitual drinker | 5 | , | 18.5 |  | 4 | , | 19.0 |  | 6 | , | 11.8 |  | 10 | , | 14.5 |  | 9 | , | 8.7 |  | 20 | , | 10.1 |  | 41 | , | 13.8 |  | 22 | , | 9.0 |  | 2 | , | 4.9 | 0.101 | b |
|  | Occasional drinker | 4 | , | 14.8 |  | 3 | , | 14.3 |  | 5 | , | 9.8 |  | 16 | , | 23.2 |  | 19 | , | 18.3 |  | 31 | , | 15.7 |  | 57 | , | 19.1 |  | 44 | , | 18.0 |  | 6 | , | 14.6 | 0.553 | b |
|  | Now abstainer | 0 | , | 0.0 |  | 0 | , | 0.0 |  | 0 | , | 0.0 |  | 1 | , | 1.4 |  | 1 | , | 1.0 |  | 1 | , | 0.5 |  | 17 | , | 5.7 |  | 10 | , | 4.1 |  | 2 | , | 4.9 | **0.002** | b |
|  | Non-drinker | 9 | , | 33.3 |  | 5 | , | 23.8 |  | 11 | , | 21.6 |  | 15 | , | 21.7 |  | 40 | , | 38.5 |  | 87 | , | 43.9 |  | 99 | , | 33.2 |  | 89 | , | 36.5 |  | 10 | , | 24.4 | 0.186 | b |
|  | Unknown | 8 |  |  |  | 22 |  |  |  | 158 |  |  |  | 73 |  |  |  | 355 |  |  |  | 1440 |  |  |  | 2172 |  |  |  | 2369 |  |  |  | 396 |  |  |  |  |
| H. pylori infection status | |  |  |  |  |  |  |  |  |  |  |  |  |  |  |  |  |  |  |  |  |  |  |  |  |  |  |  |  |  |  |  |  |  |  |  |  |  |
|  | Negative | 7 | , | 28.0 |  | 1 | , | 4.5 |  | 25 | , | 27.5 |  | 26 | , | 29.2 |  | 108 | , | 28.6 |  | 334 | , | 22.9 |  | 514 | , | 22.4 |  | 556 | , | 22.6 |  | 75 | , | 18.5 | **0.015** | b |
|  | Successful eradication | 7 | , | 28.0 |  | 7 | , | 31.8 |  | 25 | , | 27.5 |  | 16 | , | 18.0 |  | 70 | , | 18.5 |  | 299 | , | 20.5 |  | 436 | , | 19.0 |  | 487 | , | 19.8 |  | 86 | , | 21.2 | 0.367 | b |
|  | Failure eradication | 0 | , | 0.0 |  | 1 | , | 4.5 |  | 1 | , | 1.1 |  | 1 | , | 1.1 |  | 1 | , | 0.3 |  | 8 | , | 0.5 |  | 7 | , | 0.3 |  | 6 | , | 0.2 |  | 3 | , | 0.7 | 0.099 | b |
|  | Not eradication | 0 | , | 0.0 |  | 0 | , | 0.0 |  | 1 | , | 1.1 |  | 1 | , | 1.1 |  | 9 | , | 2.4 |  | 25 | , | 1.7 |  | 59 | , | 2.6 |  | 46 | , | 1.9 |  | 7 | , | 1.7 | 0.650 | b |
|  | Uninspected | 11 | , | 44.0 |  | 13 | , | 59.1 |  | 34 | , | 37.4 |  | 35 | , | 39.3 |  | 131 | , | 34.7 |  | 543 | , | 37.2 |  | 941 | , | 40.9 |  | 1055 | , | 42.9 |  | 176 | , | 43.5 | **0.001** | b |
|  | Others | 0 | , | 0.0 |  | 0 | , | 0.0 |  | 5 | , | 5.5 |  | 10 | , | 11.2 |  | 59 | , | 15.6 |  | 252 | , | 17.2 |  | 342 | , | 14.9 |  | 311 | , | 12.6 |  | 58 | , | 14.3 | 0.652 | b |
|  | Unknown | 10 |  |  |  | 21 |  |  |  | 118 |  |  |  | 53 |  |  |  | 81 |  |  |  | 177 |  |  |  | 171 |  |  |  | 152 |  |  |  | 32 |  |  |  |  |
| Hiatal hernia | | 11 | , | 31.4 |  | 14 | , | 32.6 |  | 60 | , | 28.7 |  | 56 | , | 39.4 |  | 106 | , | 23.1 |  | 439 | , | 26.8 |  | 648 | , | 26.2 |  | 697 | , | 26.7 |  | 107 | , | 24.5 | 0.121 | b |
| Reflux esophagitis | | 26 | , | 74.3 |  | 33 | , | 76.7 |  | 125 | , | 59.8 |  | 101 | , | 71.1 |  | 159 | , | 34.6 |  | 562 | , | 34.3 |  | 826 | , | 33.4 |  | 851 | , | 32.6 |  | 156 | , | 35.7 | **0.000** | b |
| Atrophic gastritis | | 13 | , | 37.1 |  | 12 | , | 27.9 |  | 51 | , | 24.4 |  | 28 | , | 19.7 |  | 146 | , | 31.8 |  | 429 | , | 26.2 |  | 616 | , | 24.9 |  | 662 | , | 25.3 |  | 120 | , | 27.5 | 0.348 | b |
| Gastric ulcer | | 0 | , | 0.0 |  | 3 | , | 7.0 |  | 8 | , | 3.8 |  | 4 | , | 2.8 |  | 19 | , | 4.1 |  | 45 | , | 2.7 |  | 74 | , | 3.0 |  | 59 | , | 2.3 |  | 6 | , | 1.4 | **0.010** | b |
| Duodenal ulcer | | 6 | , | 17.1 |  | 1 | , | 2.3 |  | 17 | , | 8.1 |  | 4 | , | 2.8 |  | 24 | , | 5.2 |  | 106 | , | 6.5 |  | 130 | , | 5.3 |  | 104 | , | 4.0 |  | 29 | , | 6.6 | **0.007** | b |
| Eosinophilic esophagitis | | 35 | , | 100.0 |  | 43 | , | 100.0 |  | 209 | , | 100.0 |  | 142 | , | 100.0 |  | 459 | , | 100.0 |  | 1638 | , | 100.0 |  | 2470 | , | 100.0 |  | 2613 | , | 100.0 |  | 437 | , | 100.0 | － | b |

Data: n, %; mean ± standard deviation.

P-value: a, linear contrast test by analysis of variance; b, Cochran–Armitage test.

2023* The 2023 survey will be conducted over a 3-month period.

※Unknown and unlisted answers were excluded from the test.

Table S2-B. Trend analysis: non-eosinophilic esophagitis eosinophilic gastrointestinal diseases (non-EoE EGID)

|  |  | 2015 | | |  | | 2016 | | | |  | | 2017 | | | |  | | 2018 | | | |  | | 2019 | | | |  | | 2020 | | | |  | | 2021 | | | |  | | 2022 | | | |  | | 2023年 | | | | P-value | |  | |  |
| --- | --- | --- | --- | --- | --- | --- | --- | --- | --- | --- | --- | --- | --- | --- | --- | --- | --- | --- | --- | --- | --- | --- | --- | --- | --- | --- | --- | --- | --- | --- | --- | --- | --- | --- | --- | --- | --- | --- | --- | --- | --- | --- | --- | --- | --- | --- | --- | --- | --- | --- | --- | --- | --- | --- | --- | --- | --- |
|  |  | (n=4) | | |  | | (n=14) | | | |  | | (n=71) | | | |  | | (n=22) | | | |  | | (n=131) | | | |  | | (n=465) | | | |  | | (n=695) | | | |  | | (n=612) | | | |  | | (n=92) | | | | for trend | |  | |  |
| Age | | 41.0 | ± | 8.8 | |  | | 45.4 | ± | 18.8 | |  | | 48.5 | ± | 17.2 | |  | | 46.7 | ± | 23.2 | |  | | 50.0 | ± | 19.8 | |  | | 49.9 | ± | 19.2 | |  | | 51.0 | ± | 19.3 | |  | | 53.0 | ± | 18.5 | |  | | 54.0 | ± | 19.7 | | **0.050** | | a | |
| Sex | |  |  |  | |  | |  |  |  | |  | |  |  |  | |  | |  |  |  | |  | |  |  |  | |  | |  |  |  | |  | |  |  |  | |  | |  |  |  | |  | |  |  |  | | 0.369 | | b | |
|  | Male | 3 | , | 75.0 | |  | | 6 | , | 42.9 | |  | | 43 | , | 60.6 | |  | | 12 | , | 54.5 | |  | | 68 | , | 51.9 | |  | | 241 | , | 51.9 | |  | | 369 | , | 53.1 | |  | | 308 | , | 50.3 | |  | | 51 | , | 55.4 | |  | |  | |
|  | Female | 1 | , | 25.0 | |  | | 8 | , | 57.1 | |  | | 28 | , | 39.4 | |  | | 10 | , | 45.5 | |  | | 63 | , | 48.1 | |  | | 223 | , | 48.1 | |  | | 326 | , | 46.9 | |  | | 304 | , | 49.7 | |  | | 41 | , | 44.6 | |  | |  | |
|  | Others | 0 | , | 0.0 | |  | | 0 | , | 0.0 | |  | | 0 | , | 0.0 | |  | | 0 | , | 0.0 | |  | | 0 | , | 0.0 | |  | | 0 | , | 0.0 | |  | | 0 | , | 0.0 | |  | | 0 | , | 0.0 | |  | | 0 | , | 0.0 | |  | |  | |
|  | Unknown | 0 |  |  | |  | | 0 |  |  | |  | | 0 |  |  | |  | | 0 |  |  | |  | | 0 |  |  | |  | | 1 |  |  | |  | | 0 |  |  | |  | | 0 |  |  | |  | | 0 |  |  | |  | |  | |
| Smoking history | |  |  |  | |  | |  |  |  | |  | |  |  |  | |  | |  |  |  | |  | |  |  |  | |  | |  |  |  | |  | |  |  |  | |  | |  |  |  | |  | |  |  |  | |  | |  | |
|  | Current smoker | 0 | , | 0.0 | |  | | 1 | , | 8.3 | |  | | 4 | , | 18.2 | |  | | 1 | , | 11.1 | |  | | 0 | , | 0.0 | |  | | 6 | , | 8.8 | |  | | 11 | , | 13.9 | |  | | 6 | , | 9.1 | |  | | 0 | , | 0.0 | | 0.783 | | b | |
|  | Former Smoker | 1 | , | 33.3 | |  | | 2 | , | 16.7 | |  | | 8 | , | 36.4 | |  | | 1 | , | 11.1 | |  | | 3 | , | 17.6 | |  | | 19 | , | 27.9 | |  | | 18 | , | 22.8 | |  | | 13 | , | 19.7 | |  | | 1 | , | 11.1 | | 0.344 | | b | |
|  | Non-smoker | 2 | , | 66.7 | |  | | 9 | , | 75.0 | |  | | 10 | , | 45.5 | |  | | 7 | , | 77.8 | |  | | 14 | , | 82.4 | |  | | 43 | , | 63.2 | |  | | 50 | , | 63.3 | |  | | 47 | , | 71.2 | |  | | 8 | , | 88.9 | | 0.306 | | b | |
|  | Unknown | 1 |  |  | |  | | 2 |  |  | |  | | 49 |  |  | |  | | 13 |  |  | |  | | 114 |  |  | |  | | 397 |  |  | |  | | 616 |  |  | |  | | 546 |  |  | |  | | 83 |  |  | |  | |  | |
| History of alcohol | |  |  |  | |  | |  |  |  | |  | |  |  |  | |  | |  |  |  | |  | |  |  |  | |  | |  |  |  | |  | |  |  |  | |  | |  |  |  | |  | |  |  |  | |  | |  | |
|  | Habitual drinker | 0 | , | 0.0 | |  | | 1 | , | 8.3 | |  | | 4 | , | 19.0 | |  | | 1 | , | 11.1 | |  | | 1 | , | 5.9 | |  | | 17 | , | 25.4 | |  | | 12 | , | 15.4 | |  | | 7 | , | 10.8 | |  | | 3 | , | 33.3 | | 0.727 | | b | |
|  | Non-habitual drinker | 2 | , | 66.7 | |  | | 4 | , | 33.3 | |  | | 2 | , | 9.5 | |  | | 1 | , | 11.1 | |  | | 2 | , | 11.8 | |  | | 8 | , | 11.9 | |  | | 13 | , | 16.7 | |  | | 7 | , | 10.8 | |  | | 0 | , | 0.0 | | 0.064 | | b | |
|  | Occasional drinker | 1 | , | 33.3 | |  | | 2 | , | 16.7 | |  | | 5 | , | 23.8 | |  | | 2 | , | 22.2 | |  | | 4 | , | 23.5 | |  | | 8 | , | 11.9 | |  | | 9 | , | 11.5 | |  | | 10 | , | 15.4 | |  | | 1 | , | 11.1 | | 0.161 | | b | |
|  | Now abstainer | 0 | , | 0.0 | |  | | 0 | , | 0.0 | |  | | 0 | , | 0.0 | |  | | 0 | , | 0.0 | |  | | 1 | , | 5.9 | |  | | 5 | , | 7.5 | |  | | 4 | , | 5.1 | |  | | 5 | , | 7.7 | |  | | 1 | , | 11.1 | | 0.092 | | b | |
|  | Non-drinker | 0 | , | 0.0 | |  | | 5 | , | 41.7 | |  | | 10 | , | 47.6 | |  | | 5 | , | 55.6 | |  | | 9 | , | 52.9 | |  | | 29 | , | 43.3 | |  | | 40 | , | 51.3 | |  | | 36 | , | 55.4 | |  | | 4 | , | 44.4 | | 0.216 | | b | |
|  | Unknown | 1 |  |  | |  | | 2 |  |  | |  | | 50 |  |  | |  | | 13 |  |  | |  | | 114 |  |  | |  | | 398 |  |  | |  | | 617 |  |  | |  | | 547 |  |  | |  | | 83 |  |  | |  | |  | |
| H. pylori infection status | |  |  |  | |  | |  |  |  | |  | |  |  |  | |  | |  |  |  | |  | |  |  |  | |  | |  |  |  | |  | |  |  |  | |  | |  |  |  | |  | |  |  |  | |  | |  | |
|  | Negative | 1 | , | 33.3 | |  | | 1 | , | 14.3 | |  | | 11 | , | 42.3 | |  | | 5 | , | 35.7 | |  | | 23 | , | 27.4 | |  | | 76 | , | 20.3 | |  | | 132 | , | 23.3 | |  | | 86 | , | 16.2 | |  | | 10 | , | 12.8 | | **0.000** | | b | |
|  | Successful eradication | 0 | , | 0.0 | |  | | 0 | , | 0.0 | |  | | 6 | , | 23.1 | |  | | 2 | , | 14.3 | |  | | 11 | , | 13.1 | |  | | 45 | , | 12.0 | |  | | 67 | , | 11.8 | |  | | 72 | , | 13.6 | |  | | 13 | , | 16.7 | | 0.621 | | b | |
|  | Failure eradication | 0 | , | 0.0 | |  | | 0 | , | 0.0 | |  | | 0 | , | 0.0 | |  | | 0 | , | 0.0 | |  | | 0 | , | 0.0 | |  | | 2 | , | 0.5 | |  | | 5 | , | 0.9 | |  | | 2 | , | 0.4 | |  | | 0 | , | 0.0 | | 0.934 | | b | |
|  | Not eradication | 0 | , | 0.0 | |  | | 0 | , | 0.0 | |  | | 0 | , | 0.0 | |  | | 0 | , | 0.0 | |  | | 4 | , | 4.8 | |  | | 16 | , | 4.3 | |  | | 14 | , | 2.5 | |  | | 11 | , | 2.1 | |  | | 2 | , | 2.6 | | 0.353 | | b | |
|  | Uninspected | 2 | , | 66.7 | |  | | 6 | , | 85.7 | |  | | 8 | , | 30.8 | |  | | 7 | , | 50.0 | |  | | 30 | , | 35.7 | |  | | 166 | , | 44.3 | |  | | 234 | , | 41.3 | |  | | 241 | , | 45.4 | |  | | 37 | , | 47.4 | | 0.459 | | b | |
|  | Others | 0 | , | 0.0 | |  | | 0 | , | 0.0 | |  | | 1 | , | 3.8 | |  | | 0 | , | 0.0 | |  | | 16 | , | 19.0 | |  | | 70 | , | 18.7 | |  | | 114 | , | 20.1 | |  | | 119 | , | 22.4 | |  | | 16 | , | 20.5 | | **0.004** | | b | |
|  | Unknown | 1 |  |  | |  | | 7 |  |  | |  | | 45 |  |  | |  | | 8 |  |  | |  | | 47 |  |  | |  | | 90 |  |  | |  | | 129 |  |  | |  | | 81 |  |  | |  | | 14 |  |  | |  | |  | |
| Hiatal hernia | | 1 | , | 25.0 | |  | | 2 | , | 14.3 | |  | | 13 | , | 18.3 | |  | | 4 | , | 18.2 | |  | | 22 | , | 16.8 | |  | | 69 | , | 14.8 | |  | | 104 | , | 15.0 | |  | | 77 | , | 12.6 | |  | | 13 | , | 14.1 | | 0.097 | | b | |
| Reflux esophagitis | | 1 | , | 25.0 | |  | | 4 | , | 28.6 | |  | | 16 | , | 22.5 | |  | | 5 | , | 22.7 | |  | | 21 | , | 16.0 | |  | | 98 | , | 21.1 | |  | | 107 | , | 15.4 | |  | | 108 | , | 17.6 | |  | | 18 | , | 19.6 | | 0.151 | | b | |
| Atrophic gastritis | | 1 | , | 25.0 | |  | | 1 | , | 7.1 | |  | | 10 | , | 14.1 | |  | | 3 | , | 13.6 | |  | | 20 | , | 15.3 | |  | | 78 | , | 16.8 | |  | | 132 | , | 19.0 | |  | | 122 | , | 19.9 | |  | | 16 | , | 17.4 | | 0.061 | | b | |
| Gastric ulcer | | 0 | , | 0.0 | |  | | 0 | , | 0.0 | |  | | 2 | , | 2.8 | |  | | 2 | , | 9.1 | |  | | 7 | , | 5.3 | |  | | 18 | , | 3.9 | |  | | 24 | , | 3.5 | |  | | 15 | , | 2.5 | |  | | 3 | , | 3.3 | | 0.262 | | b | |
| Duodenal ulcer | | 1 | , | 25.0 | |  | | 0 | , | 0.0 | |  | | 6 | , | 8.5 | |  | | 2 | , | 9.1 | |  | | 4 | , | 3.1 | |  | | 24 | , | 5.2 | |  | | 36 | , | 5.2 | |  | | 16 | , | 2.6 | |  | | 4 | , | 4.3 | | **0.029** | | b | |
| Eosinophilic gastritis and duodenitis | | 3 | , | 75.0 | |  | | 8 | , | 57.1 | |  | | 55 | , | 77.5 | |  | | 13 | , | 59.1 | |  | | 96 | , | 73.3 | |  | | 352 | , | 75.7 | |  | | 528 | , | 76.0 | |  | | 482 | , | 78.8 | |  | | 65 | , | 70.7 | | 0.164 | | b | |
| Eosinophilic enteritis and eosinophilic colitis | | 4 | , | 100.0 | |  | | 11 | , | 78.6 | |  | | 53 | , | 74.6 | |  | | 16 | , | 72.7 | |  | | 82 | , | 62.6 | |  | | 301 | , | 64.7 | |  | | 436 | , | 62.7 | |  | | 325 | , | 53.1 | |  | | 49 | , | 53.3 | | **0.000** | | b | |

Data: n, %; mean ± standard deviation.

P-value: a, linear contrast test by analysis of variance; b, Cochran–Armitage test.

2023* The 2023 survey will be conducted over a 3-month period.

※Unknown and unlisted answers were excluded from the test.
